# Maxwellian Eye Fixation during Natural Scene Perception

**DOI:** 10.1100/2012/956340

**Published:** 2012-11-25

**Authors:** Jean Duchesne, Vincent Bouvier, Julien Guillemé, Olivier A. Coubard

**Affiliations:** ^1^Laboratoire Paysage, Agrocampus Ouest, 2 rue André Le Nôtre, 49000 Angers, France; ^2^Laboratoire de Psychologie, Université d'Angers, 11 boulevard Lavoisier, 49000 Angers, France; ^3^The Neuropsychological Laboratory, CNS-Fed, 39 rue Meaux, 75019 Paris, France

## Abstract

When we explore a visual scene, our eyes make saccades to jump rapidly from one area to another and fixate regions of interest to extract useful information. While the role of fixation eye movements in vision has been widely studied, their random nature has been a hitherto neglected issue. Here we conducted two experiments to examine the Maxwellian nature of eye movements during fixation. In Experiment 1, eight participants were asked to perform free viewing of natural scenes displayed on a computer screen while their eye movements were recorded. For each participant, the probability density function (PDF) of eye movement amplitude during fixation obeyed the law established by Maxwell for describing molecule velocity in gas. Only the mean amplitude of eye movements varied with expertise, which was lower in experts than novice participants. In Experiment 2, two participants underwent fixed time, free viewing of natural scenes and of their scrambled version while their eye movements were recorded. Again, the PDF of eye movement amplitude during fixation obeyed Maxwell's law for each participant and for each scene condition (normal or scrambled). The results suggest that eye fixation during natural scene perception describes a random motion regardless of top-down or of bottom-up processes.

## 1. Introduction

In the visual exploration of a 2D scene like a photograph or a painting, the eyes fixate regions of interest to extract useful information and make large saccades to jump from one region to another. During fixation, the eyes continue to move through tremor, drifts, and microsaccades. Little is known about tremor [1–4] and drifts [5–7] due to limitations in recording systems. In contrast, microsaccades have been widely studied and their critical role in vision is being progressively uncovered (for reviews see [8, 9]).

 Though until the late 1970s [10] microsaccades were generally thought to be uncritical for vision, several studies did show microsaccades to correct random intersaccadic drifts [11], counteract retinal fatigue [12, 13], prevent visual fading [14–16], enable low-contrast discrimination [17], enhance stereoscopic hyperacuity [18], or enhance fine spatial detail [19], while tremor and drifts were still believed to be uncritical for vision [20]. In the last fifteen years, neurophysiological studies on microsaccade-induced neural activity have corroborated the idea that microsaccades refresh retinal images to prevent fading, and have reopened the debate on the role of all fixational eye movements in vision, including tremor and drifts [8, 21, 22].

 During the free viewing of a scene, visually-guided saccades depend on bottom-up processes induced by stimulus properties and on top-down influences inherent in knowledge and expectations. Though involuntary and non-conscious, fixational eye movements can also be influenced by cognitive demands [23–27]. So far, except for a few reports suggesting that fixational eye movements may [5–7, 11, 28–31] or may not be random [13, 32–35], any formalization of the random nature of eye fixation has been almost neglected. To our knowledge, only Engbert and Kliegl [30] examined this issue by showing different eye fixation behaviors depending on different time scales. In the present study, our aim was to test the hypothesis of a random generation of eye fixation. Specifically, we examined whether fixational eye movements—tremor, drifts, and microsaccades taken as a whole—showed a random motion during spontaneous visual perception as do molecules in a gas [36] or particles in a fluid [37].

 To achieve this goal, we conducted two experiments, which are presented and discussed in turn. For both experiments, we built the probability density function (PDF) of fixational eye movement amplitude, that is, the distances covered by the eyes during fixation, and compared the experimental distribution to a theoretical PDF based on the Maxwell law [36, 38, 39]. Both experiments involved a natural scene perception task to induce spontaneous and active vision as much as possible. In addition, Experiment 1 tested the influence of expertise (top-down processes) on eye fixation randomness by contrasting novice versus expert participants in perceiving natural scenes. Little is known about the effect of expertise in scene perception and the behavior in fixational eye movements, while it is established that high-attentional demand tends to suppress microsaccades [10, 23, 24, 40]. On the other hand, Experiment 2 explored the influence of stimulus properties (bottom-up processes) on eye fixation randomness by contrasting meaningful (original) versus meaningless (scrambled) scenes. Should eye fixation prove to be random in natural scene perception, the experimental PDF was expected to fit the Maxwellian PDF regardless of internal (Exp. 1) or external (Exp. 2) contingencies.

## 2. Experiment 1

### 2.1. Materials and Methods

#### 2.1.1. Participants

Eight healthy adults, 5 women and 3 men aged 28.7 ± 7.8 years (range = 22.0–44.4 yrs), took part in the study. All participants had normal vision and were unaware of the goal of the experiment.

 Three participants (1, 3, and 4) were novice to natural scene perception, whereas 4 participants (2, 5, 7 and 8) were experts: participants 2 and 5 were landscape architects; participant 7 was a designer, and participant 8 held a Ph.D. in ecology. Neither a novice nor an expert subject, participant 6 was a postgraduate student in landscape architecture.

#### 2.1.2. Ethics Statement

The study adhered to the tenets of the Declaration of Helsinki. Our study was approved by the University of Angers Ethics Committee. All participants gave their verbal consent to participate in the study; this consent procedure was approved by the local ethics committee (University of Angers, France).

#### 2.1.3. Stimuli

**Figure 1:**
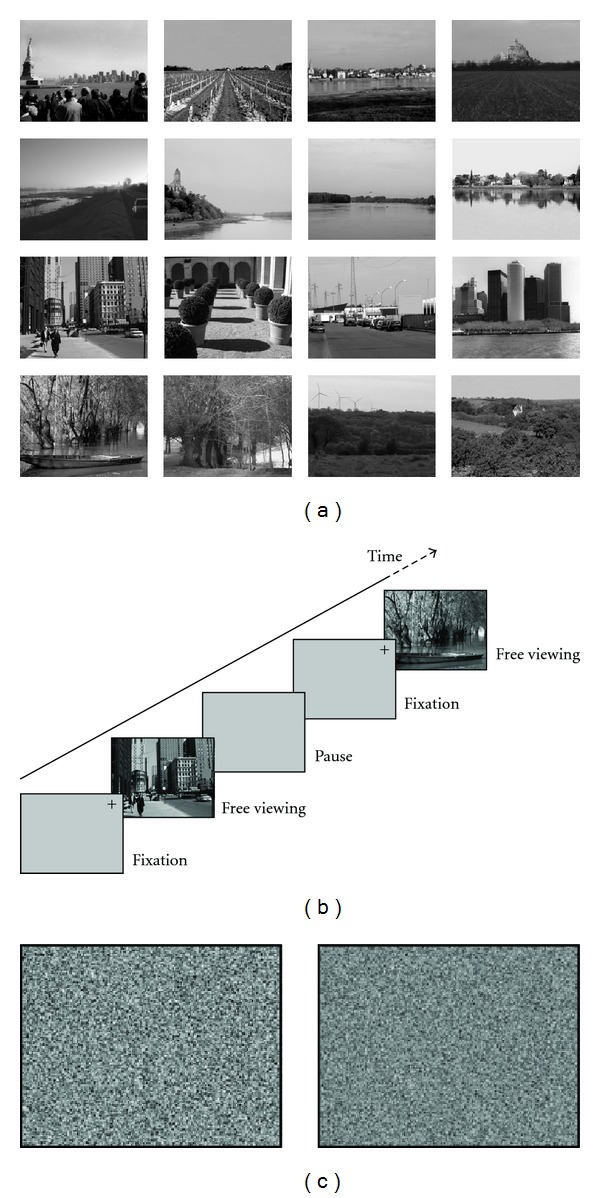
(a) Black-and-white photographs of natural scenes used as stimuli. (b) Time course of a trial in Exp. 1: after a 3-s fixation period, the stimulus appeared for free-time visual exploration before participants closed stimulus exposure by a click, then after a pause the next trial started. In Exp. 2, stimulus exposure was always 9 s and there was no pause between trials. (c) Scrambled version of scenes in Exp. 2.

Sixteen black-and-white photographs (2000*1598 pixels, 256 gray-scales) of natural scenes were taken by VB mostly in the French region of the Pays de la Loire (see Figure 1(a)). The luminance of the scenes was 121.2 ± 31.0 on a 256-gray-level scale.

 Stimuli were displayed full screen on an NEC monitor (Japan; 21 inch, 1280*1024-pixel definition, 60 Hz refresh rate) located at 1 m from participants' eyes, such that stimuli covered, respectively, 22.3° and 17.0° of visual angle horizontally and vertically.

#### 2.1.4. Apparatus

We used a faceLAB video device (Seeing Machines, Australia). A computer controlled a stereo-head, which was mounted on a tripod below the stimulation screen and held two cameras, one for each eye. Eye tracking was performed binocularly using faceLAB 4.2.2 software (Seeing Machines, Australia) in Precision Gaze configuration for optimal gaze quality level. The sample frequency was 62.5 Hz. Typical static accuracy of gaze direction was 0.5–1°. Another computer controlled the stimulation through an external screen using Gaze Tracker 05.02.03 software (Seeing Machines, Australia).

#### 2.1.5. Procedure

The experiment was conducted in a dark room; the luminance of the background was 0 cd·m^−2^. The center of the screen was adjusted at eye level and in the median plane. Calibration was performed through six steps: pupil calibration, face point reference, tracking parameters, ocular parameters, gaze calibration, and screen calibration. Gaze calibration was done using a target randomly moving on a 9-point grid that participants had to fixate as accurately as possible.

 Experiment started immediately after calibration. There were 16 trials corresponding to the 16 stimuli. The order of presentation of stimuli was random. The time course of each trial is illustrated in Figure 1(b) and was as follows. First, a fixation cross appeared for 3 s in the upper right corner of a grey screen. Then, the stimulus was displayed for the time taken by the participant for free visual exploration during which eye movements were recorded. The participant clicked on the mouse when he finished his exploration, which caused the disappearance of the scene. After a pause, he clicked again for the next trial to start. Participants were instructed to look at the scene as spontaneously as possible.

#### 2.1.6. Data Analysis

**Figure 2:**
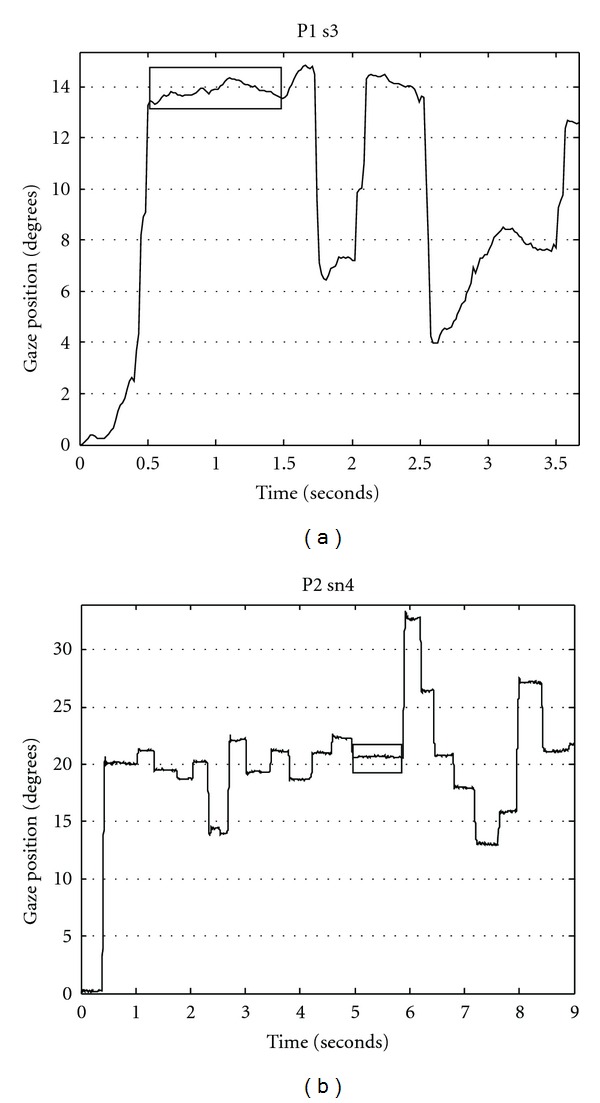
Eye fixation of participant 1 in Exp. 1 (a) and of participant 2 in Exp. 2 (b). For each graph, we show the gaze position signal, which was the average of the two eye position signals in Exp. 1 and one eye position signal in Exp. 2 (in deg) as a function of time (in s). Examples of periods of fixation are emphasized by boxes.

The 16 scenes were pooled together within each participant as we were not interested in between-scene differences. This resulted in 8 series of data (corresponding to 8 participants) which we analyzed using Gaze Tracker 05.02.03 software (Seeing Machines, Australia). Only periods of fixation were kept for further analysis. Eye fixation was determined using a sliding window algorithm operating over the set of gaze points: for each window we set two parameters (100 ms for minimum fixation duration and 42.6 arcmin for maximum fixation amplitude) to decide whether or not fixation had occurred [8].  Figure 2(a) illustrates periods of fixation.

 Using home-made scripts under Matlab 7.0 (The MathWorks, US), we then calculated the distances from the positions of the eyes in *x* and *y* planes using the Pythagorean theorem, and built the experimental and theoretical distributions. The experimental distribution was the frequency histogram of distances (i.e., eye movement amplitude during fixation), and the theoretical distribution was given by Maxwell's algorithm as described in (25) of the following section.

#### 2.1.7. Maxwell's Law

To test the hypothesis of a random distribution of eye movements during fixation, we first determined the probability density function (PDF) of eye movement amplitudes during fixation. We hypothesized an analogy between the statistical equilibrium of molecule velocity in gas and eye movement velocity during fixation. The distribution of molecule velocity in gas was formalized by Maxwell in 1859 in a statistical physics law that has since then been extensively supported by experimental data [36, 38, 39]. Though Maxwell is especially known for his findings in electromagnetism, he brought a significant contribution in thermodynamic statistics, less known but on which we focus here. The location and speed of molecules change when they collide but the distribution law remains identical, thus characterizing the gas at equilibrium: there is always the same number of molecules and their velocity ranges from *v* to *v* + *dv*. Similarly, we posited that in 2D space the PDF of fixational eye movement amplitudes is always the same: there is always the same number of amplitudes between *r* and *r* + *dr* regardless of either the observer or the observed scene. The PDF of fixational eye movement amplitudes is also a PDF of velocities as amplitudes were covered in a constant time period. We considered a symbolic space, which we called distance space. Here, our use of the term “distance” must be understood as referring to the amplitude of the movement of the eyes during fixation. In that distance space, we considered an elementary area *dxdy* around a given point *M*. That area contained a certain number *d* ^2^ *N* of distance vectors that ended inside this area. If the total number of distance vectors was *N*, we could define a function *F*(*x*, *y*) = (*d* ^2^ *N*/*N*)/*dxdy*, and if we imposed *dx* and *dy* to tend to zero, the function *NF*(*x*, *y*) represents the density of points in the sense of the geometrical probability, which corresponded to the distance in the symbolic space. If we could find the function *F*, the distance distribution could be determined.

The previous equation was rewritten as

(1) $$\begin{array}{c} \frac{d^{2}N}{N}=F\left(x,y\right)dxdy. \end{array}$$

Since the number of distance vectors *N* was constant, the normalization condition gave:

(2) $$\begin{array}{c} \iint d^{2}N=N\;\text{that}\;\;\text{is},\;\iint \;F\left(x,y\right)dx\;dy=1. \end{array}$$

The double integral was calculated between −*∞* and +*∞* in order to include all possible values of both *x* and *y*. For example, the number of distance vectors whose *x* component was between *x* and

(3) $$\begin{array}{c} x+dx\;\text{was}\;\;\frac{dN_{x}}{N}=dx\int_{-\infty }^{+\infty }dyF\left(x,y\right). \end{array}$$

Following Maxwell's method to determine molecule velocity distribution, we put forward two hypotheses: (i) the two components *x* and *y* have two independent distribution laws; this assumption is true only if the distance vector set occurs at random; (ii) the distribution of distance vectors in the distance space is isotropic. These two assumptions meant that the function *F*(*x*, *y*) took the following form:

(4) $$\begin{array}{c} F\left(x,y\right)=f\left(x\right)f\left(y\right). \end{array}$$

In other words it was the product of two identical functions *f* each of them depending on only one coordinate. Let us emphasize the fact that this was the consequence of the random choice of distance vectors. The second hypothesis was implicitly taken into account in (4) but it also implied that the point density around the origin 0 of the distance space had to respect a spherical symmetry. Another way to express this property was to say that when one moves on the circle of equation

(5) $$\begin{array}{c} x^{2}+y^{2}=C, \end{array}$$

where *C* was any constant, the function *F* had to remain constant: *F*(*x*, *y*) = *K* where *K* was any constant.

These two hypotheses were sufficient to find the distance distribution function.

Equation (5) above, which expressed the isotropy condition, was rewritten as follows:

(5^′^) $$\begin{array}{cc} & xdx+ydy=0. \end{array}$$

This led to

(6) $$\begin{array}{c} \frac{\partial F}{\partial x}dx+\frac{\partial F}{\partial y}dy=0. \end{array}$$

If we noticed after (4) that (1/*F*)(∂*F*/∂*x*) = (1/*f*(*x*))(*df*(*x*)/*dx*), we could rewrite the relation (6) as follows:

(7) $$\begin{array}{c} \frac{1}{f\left(x\right)}\frac{df\left(x\right)}{dx}dx+\frac{1}{f\left(y\right)}\frac{df\left(y\right)}{dy}dy=0. \end{array}$$

Let us recall that in (7) the differentials *dx* and *dy* were not independent since they were linked by (6).

Applying Lagrange's multiplier method to ((5′)) and (7) we obtain

(8) $$\begin{array}{c} \left[\lambda x+\frac{1}{f\left(x\right)}\frac{df\left(x\right)}{dx}\right]dx+\left[\lambda y+\frac{1}{f\left(y\right)}\frac{df\left(y\right)}{dy}\right]dy=0, \end{array}$$

where the Lagrange multiplier *λ* was an arbitrary constant. This equation could be satisfied only if the two coefficients of the differentials *dx* and *dy* were simultaneously equal to zero or written in another way:

(9) $$\begin{array}{c} \frac{1}{f\left(x\right)}\frac{df\left(x\right)}{dx}=\frac{d\left(\ln f\left(x\right)\right)}{dx}=-\lambda x, \end{array}$$

(10) $$\begin{array}{c} \frac{d\left(\ln f\left(y\right)\right)}{dy}=-\lambda y. \end{array}$$

After (9), the Lagrange multiplier *λ* depended only on *x*. Likewise, after (10) *λ* depended only on *y*. This implied that *λ* was constant and that the function *f* took the form

(11) $$\begin{array}{c} \ln f\left(x\right)=-\frac{\lambda }{2}x^{2}+\alpha \end{array}$$

or

(12) $$\begin{array}{c} f\left(x\right)=A\exp\left(-\frac{\lambda }{2}x^{2}\right) \end{array}$$

if we wrote *α* = ln⁡*A*.

Since the constant *λ* had to be positive we put *λ*/2 = *μ* ^2^.

So (12) became *f*(*x*) = *A*exp⁡(−*μ* ^2^ *x* ^2^).

As a consequence the function *F* in (4) in its turn became

(13) $$\begin{array}{c} F\left(x,y\right)=A^{2}\exp\left[-\mu^{2}\left(x^{2}+y^{2}\right)\right]. \end{array}$$

It depended only on *r* ^2^ = *x* ^2^ + *y* ^2^ which satisfied the isotropy condition. 

The number of distance vectors ended into the elementary area *dA*:

(14) $$\begin{array}{c} \frac{d^{2}N}{N}=A^{2}\exp\left[-\mu^{2}\left(x^{2}+y^{2}\right)\right]dx\;dy \end{array}$$

and the proportion of distance vectors between *r* and *r* + *dr* was obtained by considering the area bounded by two infinitely close circles whose radii were *r* and *r* + *dr*:

(15) $$\begin{array}{c} \frac{dN_{r}}{N}=A^{2}\exp\left(-\mu^{2}r^{2}\right)\iint_{r}^{r+dr}dx\;dy. \end{array}$$

In the latter equation, ∬_*r*_^*r*+*dr*^ *dx* *dy* = 2*π* *r* *dr* was the area taken into account. This led to

(16) $$\begin{array}{c} \frac{dN_{r}}{N}=A^{2}\exp\left(-\mu^{2}r^{2}\right)2\pi rdr. \end{array}$$

To determine the two constants *A* and *μ*, we used two additional conditions:

The first one said that the total number of distance vectors was *N*:

(17) $$\begin{array}{c} \iint d^{2}N=N. \end{array}$$

The first additional condition could be written ∫_−*∞*_^+*∞*^ *f*(*x*)*dx* = 1. That was to say that

(18) $$\begin{array}{c} A\int_{-\infty }^{+\infty }\exp\left(-\mu^{2}x^{2}\right)dx=1. \end{array}$$

On the other hand, we could say that the cumulative distance was

(19) $$\begin{array}{c} \iint rd^{2}N=Nm, \end{array}$$

where *m* was the mean distance which could be easily obtained from experimental data.

The second one reduced to a simple integral:

(20) $$\begin{array}{c} \frac{1}{N}\int_{0}^{\infty }rdN_{r}=m \end{array}$$

or, if we compared to (16):

(21) $$\begin{array}{c} 2\pi A^{2}\int_{0}^{\infty }r^{2}\exp\left(-\mu^{2}r^{2}\right)dr=m. \end{array}$$

Since the general integral form *I*_*n*_ = ∫_0_^*∞*^ *x* ^*n*^exp⁡(−*ax* ^2^)*dx* gives, respectively, for *n* = 0 and *n* = 2:

(22) $$\begin{array}{c} I_{0}=\frac{1}{2}{\left(\frac{\pi }{a}\right)}^{1/2},\;\;I_{2}=\frac{1}{4a}{\left(\frac{\pi }{a}\right)}^{1/2} \end{array}$$

and by remarking that in (18) the integral was calculated from −*∞* to +*∞*, (18) and (21) gave, respectively,

(23) $$\begin{array}{c} A=\frac{\mu }{\sqrt{\pi }},\;\;\frac{2\pi^{3/2}}{4\mu^{3}}A^{2}=m \end{array}$$

so that

(24) $$\begin{array}{c} \frac{dN_{r}}{N}=F\left(r\right)dr, \end{array}$$

where

(25) $$\begin{array}{c} F\left(r\right)=\frac{\pi }{2{\left(m\right)}^{2}}r\exp\left(-\frac{\pi }{4{\left(m\right)}^{2}}r^{2}\right). \end{array}$$

#### 2.1.8. Statistical Analysis

**Figure 3:**
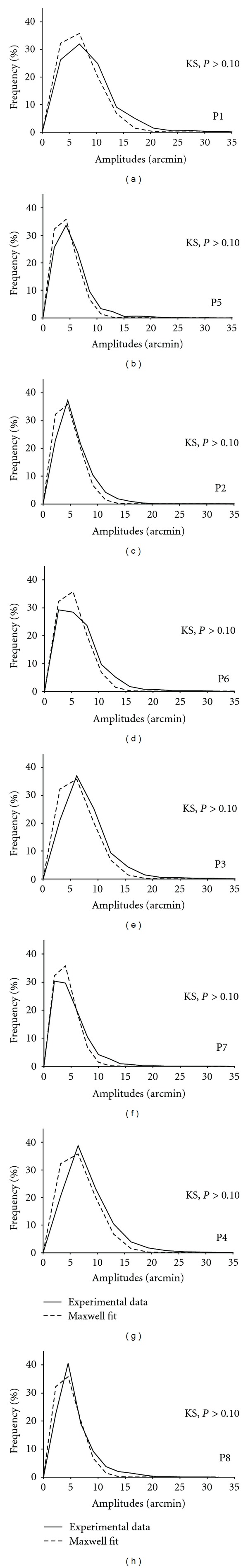
Probability density function (PDF) of eye-movement amplitudes for each participant (P1 to P8). For each graph, we show the frequency (in percentage points) as a function of amplitudes (in arcmin). The experimental PDF is shown in the full line, and Maxwell's theoretical PDF in the dotted line. For each participant, data of the 16 scenes were pooled together. Bin width was optimized using Nash and Sutcliffe's criterion and set to *m*/2 arcmin, where *m* is the mean amplitude of eye movements. KS: Kolmogorov-Smirnov (KS) two-sample test between experimental and theoretical PDFs.

Under Statistica 7.0 (StatSoft, US), experimental and theoretical distributions of eye-movement amplitude during fixation were compared using the Kolmogorov-Smirnov (KS) two-sample test for every participant. We took 95% of values as we observed for highest values a systematic gap between experimental and theoretical data, that is, we did not take into account the extreme tail of the distribution. Indeed 95% of the data in Exp. 1 spanned from 0 to 20 arcmin (see Figure 3) while the remaining 5% cover a large width from 20 to 45 arcmin. As a result classes up to 20 arcmin had a tiny size compared to the overall distribution. For that reason, we performed statistics ignoring the weakest classes as suggested by Borel et al. [41].

 To test the goodness of fit between experimental and theoretical distributions of eye movement amplitude during fixation, we took Nash and Sutcliffe's criterion from hydrology [42]. The law given by (25) in Maxwell's law section has a continuous graph. Nevertheless the graph obtained from experimental data is necessarily discontinuous. Thus, to compare the PDF of experimental data with that of simulated data, we had to choose the interval width Δ*r* of the *x*-axis in our distribution plots (see Figure 3). To achieve this goal, we used the Nash and Sutcliffe criterion or *F* value, which compares experimental and theoretical values according to the following formula:

(26) $$\begin{array}{c} F=1-\frac{\sum_{i=1}^{n}{\left(y_{\text{t}{\text{h}}_{i}}-y_{\text{ex}{\text{p}}_{i}}\right)}^{2}}{\sum_{i=1}^{n}{\left(y_{\text{t}{\text{h}}_{i}}-\bar{y_{\exp}}\right)}^{2}} \end{array}$$

in which *y*_exp_*i*__ and *y*_th_*i*__ are, respectively, experimental and theoretical values of proportion of vector distances, and $\bar{y_{\exp}}$ is the mean of experimental values. The more *F* approaches 1, the more data fit the Maxwell law. To determine the best fit, we calculated *F* values using different eye-movement amplitudes during fixation for each participant and for the group of participants.

### 2.2. Results

#### 2.2.1. Descriptive Statistics

Table 1 summarizes the viewing time per scene (in seconds), the number of samples recorded during eye fixation, the mean distance, and the SD of eye-movement amplitude (in arcmin) for each participant and for the group, and the level of expertise for each participant.

#### 2.2.2. Nash and Sutcliffe's Criterion

Table 2 shows the results for the Nash and Sutcliffe criterion using different Δ*r* from *m*/1.66 to *m*/2.66, in which *m* is the mean amplitude of eye movements during fixation. Experimental data were better simulated using a Δ*r* equal to *m*/2.5 for participants 1–4, using a Δ*r* equal to *m*/2.66 for participant 5, and using a Δ*r* equal to *m*/2 for participants 6–8. 

**Figure 4:**
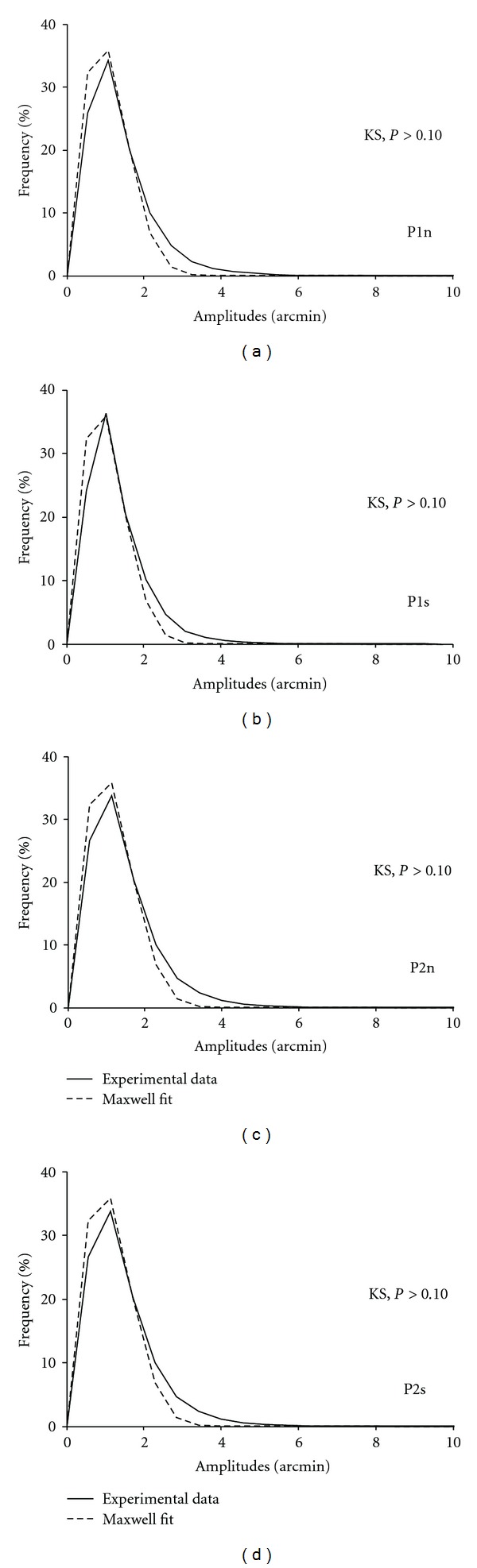
Probability density function (PDF) of eye-movement amplitudes for each participant (P1 and P2) and for each scene condition (n for normal, s for scrambled). Other notations as in Figure 3.

 For the group of participants, it was Δ*r* = *m*/2 which provided the highest mean *F* value. Thus, this Δ*r* value was selected for representing the experimental data against the simulated data in Exp. 1 (see Figure 3) and Exp. 2 (see Figure 4). This choice does not imply any basic feature of the data acquisition scheme: it emerges from the necessity to have both a high number of points and for a given point a high number of data. 

#### 2.2.3. Experimental versus Simulated Data

Figure 3 shows the PDF of experimental data and of theoretical data using Maxwell's law for each participant. There was no difference between experimental and theoretical distributions for each participant (KS, *P* > 0.10).

 We identified two groups with contrasted mean amplitude of eye movements, which was 6.13 ± 0.31 arcmin for participants 1, 3, and 4 versus 4.11 ± 0.25 arcmin for participants 2, 5, 7, and 8 (Mann-Whitney, *Z* = 2.12,  *P* < 0.05).

### 2.3. Discussion

The results of Exp. 1 suggest that eye fixation during natural scene fixation obeys Maxwell's law as the PDF of experimental data did not significantly differ from the theoretical distribution. This was true regardless of participants' expertise. The only parameter that varied was the mean amplitude of eye movements, which was higher in novice participants as compared to expert ones.

 Nevertheless, one could argue that the reported PDF of eye movement amplitude during fixation was simply a description of the noise characteristics of our eye tracker gaze estimation system. It is indeed true that the faceLAB video device is not as optimal as other systems to study eye fixation. Furthermore, Exp. 1 only used natural scenes and did not offer a comparison to controlled stimuli.

 To overcome these limitations, we undertook a second experiment in which we used a high-sample-frequency video eye tracker (1000 Hz) and added a control condition in which original scenes were filtered to produce a scrambled version of the images. Visual exploration was still free for facilitating active vision but with a fixed time equivalent to the average spontaneous viewing time of Exp. 1.

## 3. Experiment 2

### 3.1. Materials and Methods

#### 3.1.1. Participants

Two healthy men aged 40.9 ± 4.4 years (range = 37.8–44.0 yrs) participated in the study. They had corrected-to-normal vision. Both participants were novice in natural scene perception. Participants 1 and 2 were, respectively, unaware and aware of the goal of the experiment. Ethics statement was as in Exp. 1.

#### 3.1.2. Stimuli

Stimuli were the 16 natural scenes of Exp. 1. As control stimuli, a scrambled version of each scene was built using the image scrambling algorithm based on chaos theory and sorting transformation by Liu et al. [43]. Examples of scrambled stimuli are illustrated in Figure 1(c). The luminance was not different from that of original stimuli (121.2 ± 31.0 on a 256 gray-level scale; Student's *t* test, *t* < 1).

 Stimuli were displayed full screen on a Sony Trinitron monitor (Japan; 21-inch, 1280*1024-pixel definition, 100-Hz refresh rate) located at 57 cm from participants' eyes. Thus stimuli covered, respectively, 35.2° and 28.2° of visual angle horizontally and vertically.

#### 3.1.3. Apparatus

We used an EyeLink 1000 high-speed camera device (SR Research, Canada). A host computer controlled the high-speed camera used in desktop mount configuration. Eye tracking was performed monocularly using the EyeLink 1000 Host Application (SR Research, Canada). The sample frequency was 1000 Hz. Typical accuracy with the head supported was 0.25–0.5° and typical spatial resolution was <0.01° RMS. A display computer controlled the stimulation using EyeLink 1000 Host Application (SR Research, Canada).

#### 3.1.4. Procedure

The experiment was conducted in a dark room (luminance of the background was 0 cd·m^−2^). The center of the screen was adjusted at eye level and in the median plane. Head movements were minimized with chin and forehead supports. Gaze calibration was performed using a target randomly moving on a 9-point grid that participants had to fixate as accurately as possible.

 After calibration, the experiment made up of 32 trials (16 scenes and their 16 scrambled versions) started. The order of presentation of stimuli was random. The time course of each trial was as follows: a fixation cross appeared for 3 s in the upper left corner of a grey screen, then the stimulus was displayed for 9 s (corresponding to the round average of Exp. 1), then the experiment continued with the next trial. Participants were instructed to look at the scene as spontaneously as possible.

#### 3.1.5. Data Analysis

The 16 scenes were pooled together within each participant and each condition (original versus scrambled). Thus 4 series of data (2 participants times 2 conditions) were analyzed using EyeLink 1000 Host Application (SR Research, Canada). From the EyeLink Data Files provided by the software, we extracted periods of eye fixation during the 9 s visual exploration of scenes using home-made scripts under Matlab 7.0 (The MathWorks, US).  Figure 2(b) illustrates periods of fixation.

 Using home-made scripts under Matlab 7.0 (The MathWorks, US), we then calculated the distances from the positions of the eyes in *x* and *y* planes using the Pythagorean theorem, and built the experimental and theoretical distributions. The experimental distribution was the frequency histogram of distances (i.e., eye-movement amplitude during fixation). The theoretical distribution was given by Maxwell's algorithm as described in (25) of the Maxwell's law section.

#### 3.1.6. Statistical Analysis

Under Statistica 7.0 (StatSoft, US), the experimental and theoretical distributions of eye-movement amplitude during fixation were compared using the Kolmogorov-Smirnov (KS) two-sample test for every participant, using 95% of values.

### 3.2. Experiment 2: Results

#### 3.2.1. Descriptive Statistics

Table 3 presents the number of samples recorded during eye fixation, the mean distance, and the SD of distances (in arcmin) for each participant and for the group, separately for normal or scrambled scenes.

#### 3.2.2. Experimental versus Simulated Data

Figure 4 shows the PDF of experimental data and of theoretical data using Maxwell's law for each participant, separately for normal (P1n and P2n) and scrambled (P1s and P2s) scenes. There was no difference between experimental and theoretical distributions for each participant and for each scene condition (Kolmogorov-Smirnov, *P* > .10).

### 3.3. Discussion

The results of Exp. 2 using a high-sample-frequency video eye tracker suggest that eye fixation during natural scene fixation obeys Maxwell's law as the PDF of experimental data did not significantly differ from the theoretical distribution. This was true regardless of stimulus conditions, which were meaningful (original scenes) or meaningless (scrambled version of original scenes).

## 4. General Discussion

The pioneer research by Cornsweet [11] suggested that microsaccades are stochastically corrective of deviations arising from fixational drifts. Following Cornsweet, further studies investigated the randomness of drifts [5–7], whereas other studies suggested control mechanisms within the drifts [13, 32–35]. The present study sought to examine the Maxwellian nature of eye fixation applying statistical physics to the psychophysics of eye movements. Using a video eye tracker during free (Exp. 1) or fixed-time (Exp. 2) visual exploration of natural scenes, we showed that the amplitude of eye movements during fixation obeys Maxwell's law suggesting that fixational eye movements describe a motion similar to that of molecules in a gas [36] or of particles in organic and inorganic bodies [37].

 Since in Exp. 1 the sample frequency of our video eye tracker was weak (62.5 Hz), Exp. 2 used a higher-sample frequency (1000 Hz) providing a similar pattern of results. In both cases, we did not differentiate between the different subtypes of fixational eye movements, and the Maxwellian nature of eye fixation we report here concerns fixational eye movements as a whole. The fact that our experimental data obeyed Maxwell's law in both experiments corroborate the proposal that for Brownian motion the result is independent from measure frequency, as originally suggested by Perrin [44, 45]. We have exhaustively presented Maxwell's law as it is the first time, to our knowledge, that such a demonstration has been made in 2D space. We took the Nash and Sutcliffe criterion from hydrology [42] to optimize the goodness of fit between observational and simulated data. To our knowledge, it is also the first time that this criterion has been used in visual science.

 We used natural scenes as stimuli enabling us to elicit spontaneous and active vision, that is, without any instruction except spontaneously looking at the scene. As we were only interested in absolute fixational activity, we did not seek the superimposition of eye movements with visual stimuli. Exp. 1 showed two contrasted groups based on the mean amplitude of eye movements. Interestingly, the group showing the highest mean amplitude was made of novice participants (1, 3, and 4), that is of individuals without any professional experience of visual exploration of natural scenes in 2D or 3D space. In contrast, the group exhibiting the lowest mean amplitude was made up of visual experts. Indeed, participants 2 and 5 were two landscape architects, participant 7 was a designer, and participant 8 held a PhD in ecology: all were confirmed practitioners of daily visual observation and analysis of natural or artificial scenes through either photographs of scenes or landscapes in the real 3D world. We therefore suggest that the mean amplitude of eye movements during fixation of natural scenes may be a tool for measuring visual expertise. One hypothesis is that expertise may have led to fixational eye movement suppression due to higher attentional control, in line with studies showing that microsaccades are suppressed in high-acuity or high-attentional demand tasks [10, 23, 24, 40]. Interestingly between the two groups of novice versus expert subjects, participant 6, who was a postgraduate student in landscape architecture, exhibited intermediate mean amplitude of fixational eye movements of 4.93 arcmin, suggesting such a measure may be sensitive.

 Another point that needs discussion is that Exp. 1 led to a mean amplitude of eye movements during fixation equal to 5.291 arcmin for the group of eight participants, whereas Exp. 2 yielded a mean amplitude equal to 1.166 arcmin for the group of two participants. Thus, the ratio between Exp. 2 and Exp. 1 was of 5.291/1.166 = 4.538. One could argue that we should have got a ratio of 16 (i.e., 1000/62.5), with respect to the different sample frequency between Exp. 2 (1000 Hz) and Exp. 1 (62.5 Hz). However such inference would be erratic. In fact, this ratio of around 4 is an additional demonstration of the Brownian nature (i.e., random) of fixational eye movements during natural scene perception. Indeed, the mean of Brownian displacements in *a given time period* is not proportional to the number of shocks (in our study, the number of displacements) but to the square root of the number of shocks, thus $\sqrt{16}=4$ in our study [44, 46, 47]. That we obtained 4.538 and not exactly 4 may simply be explained by the fact that we manipulated not physical but physiological data, added to the fact that Exp. 2 only included two participants thus lowering statistical power. In all, we suggest that the ratio of ~4 for the mean amplitude of eye movements between the two experiments is further evidence of the random nature of eye fixation during natural scene perception. Importantly, this hypothesis does not exclude that both stochastic and deterministic structures may be hidden below the Maxwellian macrostructure of fixational eye movements taken as a whole, which would warrant further investigation using finer dynamic models (e.g., [30]).

 Why would the central nervous system randomize fixational eye movements? Recently, Engbert and Kliegl [30] applied statistical physics to microsaccades during fixation using an approach developed for analyzing human posture control [48]. In a re-examination of Cornsweet's hypothesis [11], the authors showed that during eye fixation of a stationary spot, microsaccades both produce fixation errors to enhance perception on a short time scale and reduce fixation errors and binocular disparity on a long time scale, which would weigh against a random uncontrolled movement [30]. Differences in results with the present study may be due to the different nature of the task, or more likely to the different analyses, and thus needs to be further investigated. A final issue concerns the neural underpinnings of such a random generator for fixational eye movements. It would be interesting to verify whether some neurones exhibit the Maxwell law in their firing code. Potential candidate neurones may be those of the foveal portion of the superior colliculus, which have recently been shown to play a causal role in microsaccade generation in primates [49].

 To conclude, our results show that fixational eye movements during natural scene perception obey Maxwell's law and support the fact that eye movements during fixation describe a motion similar to that of molecules in gas [36, 38, 39] or particles in organic and inorganic bodies [37]. Such a Maxwellian nature of eye fixation is robust since it is independent of top-down processes such as participants' expertise (Exp. 1) or of bottom-up processes such as those inherent in physical and semantic properties of the stimulus (Exp. 2.)

## Figures and Tables

**Table 1:** Results of Experiment  1 for each participant and for the group.

Participants	Viewing time (s)	Number of samples	Mean distance (arcmin)	SD distance (arcmin)	Expertise
1	1.48	1240	6.867	4.583	Novice
2	25.00	21322	4.586	3.212	Expert
3	5.09	4075	6.20	4.220	Novice
4	8.62	7482	6.494	4.172	Novice
5	14.42	2300	4.318	3.286	Expert
6	4.18	3597	5.249	4.061	Intermediate
7	4.33	3675	4.025	3.234	Expert
8	6.02	5266	4.585	3.463	Expert

Mean ± SD	8.64 ± 7.66	—	5.291 ± 1.089	3.779 ± 0.539	—

**Table 2:** Nash and Sutcliffe's criterion (*F* value) for each participant and for the group using different Δ*r* (*m* is the mean distance).

Participants	Δ*r* = *m*/1.66	Δ*r* = *m*/2	Δ*r* = *m*/2.5	Δ*r* = *m*/2.66
1	0.905	0.954	0.961	0.957
2	0.936	0.939	0.957	0.926
3	0.896	0.915	0.970	0.936
4	0.825	0.913	0.940	0.938
5	0.920	0.939	0.928	0.960
6	0.921	0.939	0.903	0.926
7	0.910	0.958	0.797	0.783
8	0.868	0.930	0.895	0.875

Mean ± SD	0.898 ± 0.036	0.936 ± 0.016	0.919 ± 0.056	0.913 ± 0.059

**Table 3:** Results of Experiments 2 for each participant and for the group.

Participants	Number of samples	Mean distance (arcmin)	SD distance (arcmin)	Number of samples	Mean distance (arcmin)	SD distance (arcmin)
Scene type	Normal	Normal	Normal	Scrambled	Scrambled	Scrambled
1	121228	1.080	0.836	126148	1.028	0.782
2	118195	1.252	0.959	116378	1.143	0.875

Mean ± SD	—	1.166 ± 0.122	0.898 ± 0.087	—	1.085 ± 0.081	0.829 ± 0.066

## References

[1] Eizenman M, Hallett PE, Frecker RC (1985). Power spectra for ocular drift and tremor. *Vision Research*.

[2] Michalik M (1987). Spektranalysen des okulären mikrotremors bein hirnstammfunktionsstörungen. *Zeitschrift Für Electroenzephalographie Und Elektromyographie*.

[3] Spauschus A, Marsden J, Halliday DM, Rosenberg JR, Brown P (1999). The origin of ocular microtremor in man. *Experimental Brain Research*.

[4] Ramdane-Cherif Z, Naït-Ali A, Motsch JF, Krebs MO (2004). An autoregressive (AR) model applied to eye tremor movement, clinical application in schizophrenia. *Journal of Medical Systems*.

[5] Matin L, Matin E, Pearce DG (1970). Eye movements in the dark during the attempt to maintain a prior fixation position. *Vision Research*.

[6] Liang JR, Moshel S, Zivotofsky AZ (2005). Scaling of horizontal and vertical fixational eye movements. *Physical Review E*.

[7] Mergenthaler K, Engbert R (2007). Modeling the control of fixational eye movements with neurophysiological delays. *Physical Review Letters*.

[8] Martinez-Conde S, Macknik SL, Hubel DH (2004). The role of fixational eye movements in visual perception. *Nature Reviews Neuroscience*.

[9] Rolfs M (2009). Microsaccades: small steps on a long way. *Vision Research*.

[10] Kowler E, Steinman RM (1979). Miniature saccades: eye movements that do not count. *Vision Research*.

[11] Cornsweet TN (1956). Determination of the stimuli for involuntary drifts and saccadic eye movements. *Journal of the Optical Society of America*.

[12] Ditchburn RW, Fender DH, Mayne S (1959). Vision with controlled movements of the retinal image. *The Journal of Physiology*.

[13] Nachmias J (1961). Determiners of the drift of the eye during monocular fixation. *Journal of the Optical Society of America*.

[14] Riggs LA, Ratliff F (1952). The effects of counteracting the normal movements of the eyes. *Journal of the Optical Society of America*.

[15] Ditchburn RW, Ginsborg BL (1952). Vision with a stabilized retinal image. *Nature*.

[16] Yarbus AL (1967). *Eye Movements and Vision*.

[17] Ditchburn RW, Kowler E, Steinman RM (1980). The function of small saccades: small saccades serve no useful purpose. *Vision Research*.

[18] Westheimer G (1979). The spatial sense of the eye. *Investigative Ophthalmology and Visual Science*.

[19] Rucci M, Iovin R, Poletti M, Santini F (2007). Miniature eye movements enhance fine spatial detail. *Nature*.

[20] Carpenter RHS (1988). *Of the Eyes*.

[21] Martinez-Conde S, Macknik SL, Hubel DH (2000). Microsaccadic eye movements and firing of single cells in the striate cortex of macaque monkeys. *Nature Neuroscience*.

[22] Martinez-Conde S, Macknik SL, Troncoso XG, Hubel DH (2009). Microsaccades: a neurophysiological analysis. *Trends in Neurosciences*.

[23] Winterson BJ, Collewijn H (1976). Microsaccades during finely guided visuomotor tasks. *Vision Research*.

[24] Bridgeman B, Palca J (1980). The role of microsaccades in high acuity observational tasks. *Vision Research*.

[25] Valsecchi M, Turatto M (2007). Microsaccadic response to visual events that are invisible to the superior colliculus. *Behavioral Neuroscience*.

[26] Valsecchi M, Turatto M (2009). Microsaccadic responses in a bimodal oddball task. *Psychological Research*.

[27] Hermens F, Zanker JM, Walker R (2010). Microsaccades and preparatory set: a comparison between delayed and immediate, exogenous and endogenous pro- and anti-saccades. *Experimental Brain Research*.

[28] Findlay JM (1971). Frequency analysis of human involuntary eye movement. *Kybernetik*.

[29] Findlay JM (1974). Direction perception and human fixation eye movements. *Vision Research*.

[30] Engbert R, Kliegl R (2004). Microsaccades keep the eyes’ balance during fixation. *Psychological Science*.

[31] Moshel S, Zivotofsky AZ, Jin-Rong L (2008). Persistence and phase synchronisation properties of fixational eye movements. *European Physical Journal*.

[32] Nachmias J (1959). Two-dimensional motion of the retinal image during monocular fixation. *Journal of the Optical Society of America*.

[33] Boyce PR (1967). Monocular fixation in human eye movement. *Proceedings of the Royal Society of London. Series B*.

[34] Steinman RM, Haddad GM, Skavenski AA, Wyman D (1973). Miniature eye movement: the pattern of saccades made by man during maintained fixation may be a refined but useless motor habit. *Science*.

[35] Møller F, Laursen ML, Sjølie AK (2006). The contribution of microsaccades and drifts in the maintenance of binocular steady fixation. *Graefe’s Archive for Clinical and Experimental Ophthalmology*.

[36] Reif F (1965). *Fundamentals of Statistical and Thermal Physics*.

[37] Brown R (1828). A brief account of microscopical observations made in the months of June, July and August, 1827, on the particles contained in the pollen of plants; and on the general existence of active molecules in organic and inorganic bodies. *Philosophical Magazine*.

[38] Sears FW (1959). *Thermodynamics*.

[39] Gupta MC (1991). *Statistical Thermodynamics*.

[40] Kowler E, Steinman RM (1977). The role of small saccades in counting. *Vision Research*.

[41] Borel E, Deltheil R, Huron R (1964). *Probabilités, Erreurs*.

[42] Nash JE, Sutcliffe JV (1970). River flow forecasting through conceptual models part I—a discussion of principles. *Journal of Hydrology*.

[43] Liu X, Zhang J, Zhang J, He X (2008). Image scrambling algorithm based on chaos theory and sorting transformation. *International Journal of Computer Science and Network Security*.

[44] Perrin J (1913). *Les Atomes*.

[45] Diu B, Lederer D, Roulet B (1996). *Physique Statistique*.

[46] Einstein A (1905). Über die von der molekularkinetischen theorie der wärme geforderte bewegung von in ruhenden flüssigkeiten suspendierten teilchen. *Annalen der Physik*.

[47] Mandelbrot B (1977). *Fractals: Form, Chance and Dimension*.

[48] Collins JJ, De Luca CJ (1993). Open-loop and closed-loop control of posture: a random-walk analysis of center-of-pressure trajectories. *Experimental Brain Research*.

[49] Hafed ZM, Goffart L, Krauzlis RJ (2009). A neural mechanism for microsaccade generation in the primate superior colliculus. *Science*.

